# Optimizing outcomes in intracranial ependymoma: a contemporary review

**DOI:** 10.3389/fonc.2025.1617169

**Published:** 2025-06-10

**Authors:** Zhenjiang Pan, Jing Bao, Shepeng Wei

**Affiliations:** ^1^ Department of Neurosurgery, Shidong Hospital, Yangpu District, Shanghai, China; ^2^ Shidong Hospital, University of Shanghai for Science and Technology, Shanghai, China

**Keywords:** intracranial ependymoma, molecular classification, gross total resection, radiotherapy, pediatric neuro-oncology

## Abstract

Intracranial ependymomas are glial tumors arising from the ependymal lining of the ventricular system, most commonly affecting young children (median age: 5 years), though they can occur across all age groups. Typically located in the posterior fossa, they account for fewer than 10% of pediatric central nervous system neoplasms and show a slight male predominance. Clinical symptoms vary by location, with posterior fossa tumors often causing hydrocephalus-related signs, and supratentorial lesions presenting with seizures or focal deficits. The 2021 WHO CNS5 classification integrates histologic, anatomic, and molecular features, distinguishing prognostically significant subgroups such as posterior fossa group A (PFA) and supratentorial ZFTA-fusion ependymomas. Diagnosis requires histologic confirmation, aided by MRI and cerebrospinal fluid analysis, with dissemination present in up to 10% of cases at diagnosis. Maximal safe surgical resection is the cornerstone of treatment. Children over one year with grade 2 or 3 tumors typically receive adjuvant focal radiotherapy, while chemotherapy is used to delay irradiation in infants or after subtotal resection. Disseminated disease may require craniospinal irradiation or systemic therapy. Despite multimodal treatment, prognosis remains guarded. Ten-year overall survival ranges from 50% to 75%, influenced by extent of resection, molecular subtype, and age. This review synthesizes current knowledge of ependymoma pathogenesis, classification, diagnosis, and therapy, highlighting the growing role of molecular profiling and the importance of specialized, multidisciplinary care.

## 1 Introduction

Ependymomas are glial tumors thought to originate from radial glial cells in the subventricular zone, typically adjacent to the ependymal lining of the ventricular system (1–3). They predominantly occur in the posterior fossa near the fourth ventricle or within the intramedullary spinal cord, with rare occurrences in the cerebral parenchyma outside the posterior fossa and exceptional cases outside the central nervous system. This review focuses on the clinical presentation and management of intracranial ependymomas.

Ependymomas are glial tumors that arise from cells lining the ventricular system of the brain and spinal cord. Although they represent a relatively small proportion of central nervous system (CNS) tumors overall, they pose a significant clinical challenge due to their location, potential for recurrence, and molecular heterogeneity. In pediatric populations, intracranial ependymomas account for approximately 5–10% of all primary CNS tumors, with the posterior fossa being the most common site of origin (1–4). In contrast, adults more frequently develop spinal ependymomas, although intracranial variants are not uncommon (1, 3, 5).

Historically, classification and prognostication relied heavily on histopathological grading and anatomic location. However, recent advances in molecular diagnostics have revolutionized the understanding and management of these tumors. The 2021 World Health Organization (WHO) Classification of Tumors of the Central Nervous System (CNS5) emphasizes integrated diagnoses based on a combination of histology, molecular markers, and anatomical site (6, 7). This paradigm shift has led to the recognition of distinct molecular subgroups with divergent clinical behaviors and outcomes, such as posterior fossa group A (PFA), group B (PFB), and supratentorial ZFTA- or YAP1-fused tumors (8–10).

In this review, we aim to summarize contemporary approaches to the diagnosis and treatment of intracranial ependymomas, highlighting the critical role of molecular characterization in guiding clinical decision-making. We also explore ongoing controversies, emerging therapies, and directions for future research in this evolving field.

## 2 Epidemiology

Intracranial ependymomas predominantly affect young children, with a slight male predominance (3, 4). The median age at diagnosis is 5 years, with 25–40% of cases occurring before age 2 (5–7). In adults, most cases present before age 40. Ependymomas account for less than 10% of central nervous system tumors in children and young adults but represent approximately 25% of primary spinal cord tumors (8, 9). Spinal ependymomas typically arise between ages 30 and 40 and are more prevalent in patients with NF2-related schwannomatosis. Subependymomas, often incidental or identified at autopsy, primarily affect middle-aged and older men (3, 4).

## 3 Pathology

### 3.1 Histology and classification

In the World Health Organization (WHO) classification of central nervous system (CNS) tumors, ependymal tumors are categorized by anatomic location, histology, and molecular characteristics. The 2021 revision (5th edition, CNS5) introduces several new entities and subgroups defined by molecular genetic features (3, 10).

#### 3.1.1 Ependymomas

Ependymomas may arise throughout the ventricular system and spinal canal, most commonly in the fourth ventricle and spinal cord. They are typically well circumscribed and may show calcification, hemorrhage, or cystic change. Ependymal rosettes, though not always present, are a diagnostic hallmark. Histologic variants—including papillary, clear cell, and tanycytic—lack distinct clinical relevance.

Traditionally classified as classic or anaplastic based on cellularity and mitotic activity, ependymomas have shown inconsistent correlation between histologic grade and prognosis. Some studies, such as the Children’s Oncology Group ACNS0121 phase II trial (11), suggest worse outcomes with anaplastic (grade 3) tumors, but findings are not uniform, particularly in retrospective analyses (12). Tumor heterogeneity and interobserver variability may underlie this inconsistency (13).

Reflecting improved understanding of molecular subgroups, the WHO CNS5 classification abandons the classic–anaplastic distinction and incorporates histology, location, and molecular features (10). Histologic grading remains an integral part of diagnosis and prognostication, although its predictive power is increasingly supplemented by molecular classification (14–18).

#### 3.1.2 Subependymoma

Subependymomas are rare WHO grade 1 tumors typically located in the fourth or lateral ventricles of adults (3). They have a benign histologic appearance, featuring a coarse fibrillar matrix, uniform nuclei in clusters, microcysts, and occasionally calcifications or hemorrhage.

#### 3.1.3 Myxopapillary ependymoma

Myxopapillary ependymomas arise almost exclusively in the conus medullaris and filum terminale. Molecular differences have been noted between adult and pediatric cases (19). Under the WHO CNS5 classification, they are designated as grade 2, reflecting a recurrence risk similar to conventional spinal ependymomas (3, 10, 20–23).

### 3.2 Molecular groups

Epigenomic and transcriptomic analyses have identified at least nine distinct molecular entities of ependymoma, each with unique demographics and clinical behavior (33). Further “omics” studies reveal additional heterogeneity within posterior fossa group A (PFA) (34) and group B (PFB) (35) tumors. Mapping of enhancer landscapes may provide a basis for targeted drug development across molecular subtypes (36).

Genomic alterations in ependymomas vary by anatomic site (16–18, 24–36). Comprehensive genomic and epigenetic profiling has defined distinct molecular subgroups (24, 37–39). Emerging classifications continue to evolve and require prospective validation to establish their prognostic and therapeutic relevance (40).

#### 3.2.1 Posterior fossa ependymoma group A

PFA ependymomas, an aggressive molecular subgroup, primarily affect infants and young children (2, 12, 41, 42). These tumors exhibit a CpG island methylator phenotype and transcriptional silencing of polycomb repressive complex 2 (PRC2), leading to downregulation of differentiation genes (43). Chromosomal alterations—particularly 1q gain, which is found in 15–20% of newly diagnosed cases—are associated with poor outcomes. Combined 1q gain and 6q loss occurs less frequently but may define an ultra–high-risk subgroup. These alterations are detected in up to 60% of recurrent cases (11, 12, 15, 16, 18, 24, 28, 44–46).

Loss of nuclear H3K27me3 expression—a hallmark of PFA tumor cells (32)—was first described in H3 K27-altered diffuse midline gliomas (47). Both tumor types appear to share a PRC2-inhibitory oncogenic mechanism involving peptidyl PRC2 inhibitors (48, 49). Immunohistochemical absence of H3K27me3 offers a reliable, clinically applicable marker for identifying PFA tumors (31, 50).

#### 3.2.2 Posterior fossa ependymoma group B

PFB ependymomas predominantly affect older children and adults and are linked to a more favorable prognosis (38, 41).

#### 3.2.3 Supratentorial ependymoma with ZFTA fusion

Most supratentorial ependymomas (70–80%) harbor an oncogenic fusion between ZFTA (formerly C11orf95) on chromosome 11 and a partner gene—most commonly RELA, a key effector of NF-κB signaling (51, 52). ZFTA fusions are typically identified by fluorescence *in situ* hybridization (FISH), although RNA sequencing and next-generation sequencing (NGS) are also widely used in molecular diagnostic workflows. Although retrospective studies have linked these fusions to poor prognosis (51), prospective data have challenged this association (11, 12, 53, 54), warranting caution in clinical interpretation. Additional prognostic markers are under investigation. Biallelic CDKN2A loss has emerged as a candidate marker of poor outcome in ZFTA-fusion tumors, though validation in prospective cohorts is needed (45, 55, 56).

#### 3.2.4 Supratentorial ependymoma with YAP1 fusion

YAP1 fusion tumors represent a small fraction of supratentorial ependymomas and are predominantly observed in infants. This subgroup may exhibit a more favorable prognosis compared with other ependymoma subtypes (12, 45), though prospective validation is required.

#### 3.2.5 Supratentorial ependymoma without ZFTA or YAP1 fusion

A minority of supratentorial ependymomas lack ZFTA or YAP fusions and display clinical and molecular heterogeneity, underscoring the need for larger studies to clarify their biology (57, 58).

## 4 Clinical features

### 4.1 Presenting signs and symptoms

Clinical presentation varies by tumor location:

#### 4.1.1 Increased intracranial pressure

Posterior fossa ependymomas commonly cause obstructive hydrocephalus, leading to headache, nausea, vomiting, ataxia, vertigo, and papilledema. Cranial nerve palsies—particularly of nerves VI to X—are frequent, and brainstem invasion may occur.

#### 4.1.2 Seizures and focal deficits

Supratentorial ependymomas often present with seizures or focal deficits, such as hemiparesis, due to mass effect and peritumoral edema.

#### 4.1.3 Myelopathy and radiculopathy

Spinal ependymomas cause symptoms from tract or nerve root involvement, with findings determined by tumor location along the cord.

#### 4.1.4 Leptomeningeal disease

Cerebrospinal fluid (CSF) dissemination is present in fewer than 5% of patients at diagnosis (12, 53, 59). Both infratentorial and supratentorial tumors may spread, with clinical presentations ranging from minimal symptoms to multifocal deficits involving cranial nerves or the cauda equina. Although earlier studies linked spinal seeding to higher histologic grade, recent data suggest that prognosis is more closely associated with molecular subgroup.

### 4.2 Anatomic location

The fourth ventricle is the most frequent site of intracranial ependymomas, often extending into the subarachnoid space and occasionally encasing the medulla and upper cervical cord. Supratentorial tumors may be intraventricular (typically in the lateral ventricles) or parenchymal. Ependymoma locations vary by age:

#### 4.2.1 Children

Approximately 90% of ependymomas in children are intracranial, with 75% in the posterior fossa and 10% in the spinal cord (5, 6).

#### 4.2.2 Adults

In adults, about 65% are spinal, 25% infratentorial, and 10% supratentorial (60).

## 5 Diagnosis

Diagnosis of ependymoma requires histologic confirmation but is often suspected preoperatively based on imaging, tumor location, and patient age. Because gross total resection is central to management—and appropriate for most differential diagnoses in children—diagnosis is typically made during resection. Biopsy is reserved for cases with diagnostic uncertainty or high surgical risk.

### 5.1 Neuroimaging appearance

On magnetic resonance imaging (MRI), posterior fossa ependymomas typically appear hypointense on T1-weighted and hyperintense on T2-weighted or proton density sequences, with prominent gadolinium enhancement. Extension into the foramen of Luschka is common. These tumors often obstruct the fourth or supratentorial ventricles, causing hydrocephalus; however, peritumoral edema is uncommon. Restricted diffusion may be present. Supratentorial ZFTA-fused ependymomas frequently have a large cystic component with nodular areas showing restricted diffusion (61, 62).

On computed tomography (CT), ependymomas are usually hyperdense with homogeneous enhancement and may contain cysts or calcifications. Calcifications in a fourth ventricle mass suggest ependymoma but are not pathognomonic. Subependymomas appear as nonenhancing, well-circumscribed intraventricular nodules, isodense on CT and typically isointense on T1 and hyperintense on T2 MRI sequences (63).

### 5.2 Extent of disease evaluation

All patients with suspected or confirmed ependymoma should undergo contrast-enhanced MRI of the brain and entire spine, along with cerebrospinal fluid (CSF) analysis. Spinal or leptomeningeal dissemination occurs in up to 10% of cases, though some reports suggest a lower incidence at diagnosis (12, 53, 59). CSF cytology is recommended for staging in classic, anaplastic, and myxopapillary ependymomas when imaging suggests spread.

Preoperative lumbar puncture is preferred, as postoperative samples may be contaminated by surgical debris. However, obstructive hydrocephalus often precludes lumbar puncture at presentation. In such cases, CSF sampling should be deferred for 10–14 days postoperatively to allow debris clearance (64). Although dissemination is rare, it significantly affects management and prognosis. Notably, about one-third of cases are identified solely by CSF cytology (53, 65). Given the potential for cytologic misinterpretation, a second CSF sample is advised to confirm isolated positive results.

## 6 Surgical resection

The primary treatment for suspected ependymoma is maximal safe resection.

### 6.1 Extent of resection

Ependymomas often arise in the posterior fossa, adjacent to cranial nerves and the brainstem, making resection challenging. Nonetheless, extent of resection is a key determinant of oncologic outcome and survival, underscoring the importance of optimal initial surgery. Referral to centers with pediatric oncologic neurosurgical expertise is strongly recommended. While terminology may vary, resection extent is generally classified as follows:

#### 6.1.1 Gross total resection

Achieved when postoperative MRI reveals no residual enhancing or nonenhancing tumor, and the surgeon confirms complete removal intraoperatively.

#### 6.1.2 Near total resection

Near-total resection refers to minimal residual tumor on postoperative MRI (typically <5 mm) or no visible disease, despite intraoperative evidence of tumor adherence to critical structures such as the brainstem or cranial nerves that prevents complete resection. For prognostic and therapeutic purposes, these cases are generally treated similarly to gross total resections.

#### 6.1.3 Subtotal resection

Subtotal resection—defined by residual tumor on postoperative MRI and encompassing terms such as partial or incomplete resection—has not been evaluated in randomized trials. Given the overwhelming observational evidence supporting gross total resection, randomizing patients to subtotal resection would now be considered ethically impermissible. However, observational studies consistently associate gross total resection with lower local recurrence and improved long-term survival compared to partial resection (66–69). Most deaths in prospective studies are due to local recurrence, which is often difficult to control.

Brainstem-invasive tumors present particular surgical challenges, and outcomes are poorer with incomplete resection. As a result, many centers use preradiation chemotherapy and consider second-look surgery when residual tumor appears safely resectable.

### 6.2 Complications

Common complications following posterior fossa ependymoma resection include:

#### 6.2.1 Cerebellar ataxia

Ataxia may emerge or worsen postoperatively. Injury to the lateral cerebellar hemispheres causes limb dysmetria, while midline involvement leads to gait ataxia.

#### 6.2.2 Lower cranial nerve injury

Tumors in the cerebellopontine angle can damage lower cranial nerves, resulting in hemifacial weakness, dysarthria, dysphagia, or hearing loss. Infants may experience functional recovery after resection.

#### 6.2.3 Posterior fossa syndrome

Cerebellar mutism, or posterior fossa syndrome, is a well-recognized complication, particularly when the superior or middle cerebellar peduncles are involved (70–73).

## 7 Postoperative therapy

### 7.1 Intracranial ependymoma, grade 2 or 3

Ependymomas are infiltrative primary brain tumors capable of CNS dissemination. Management typically includes maximal safe resection, followed by radiotherapy and, in some cases, chemotherapy—tailored to patient age, tumor location, and extent of resection (algorithm 1) (39, 74, 75). At present, treatment is not stratified by molecular subgroup.

**Figure 1:**
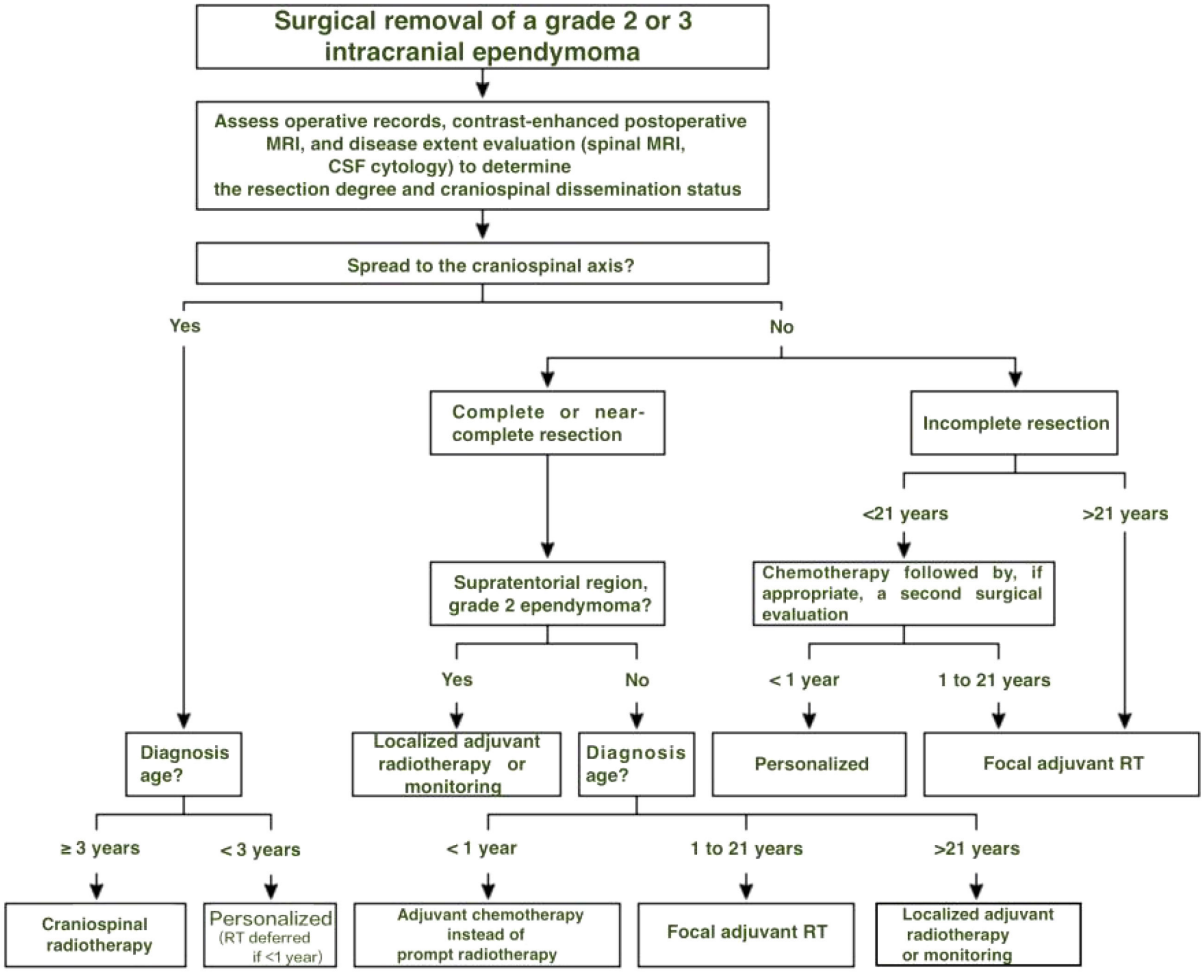
Treatment decision algorithm following surgical removal of grade 2 or 3 intracranial ependymoma. In children under 1 year, radiotherapy is generally deferred due to neurodevelopmental risks. Chemotherapy is used to delay irradiation whenever feasible, including in cases with craniospinal dissemination.

Given the complexity of care, referral to specialized centers is strongly advised, particularly for infants with subtotal resections or patients with positive cerebrospinal fluid cytology. A treatment algorithm based on age, resection extent, and craniospinal dissemination is presented in **Figure 1**.

#### 7.1.1 Gross total resection, age 1 to 21 years

Postoperative management of children older than one year with gross or near-total resection of intracranial grade 2 or 3 ependymomas continues to evolve. Radiotherapy alone remains standard in most cases (74–77). Preliminary data from the COG ACNS0831 trial suggest a possible benefit of maintenance chemotherapy after RT in selected patients, though final results are pending (78).

An exception includes children with supratentorial grade 2 tumors who achieve gross total resection; these patients may have favorable outcomes with observation alone. This approach was evaluated in one arm of the ACNS0831 trial, with outcomes not yet reported.

##### 7.1.1.1 Adjuvant focal RT

In children over 1 year of age with grade 2 or 3 ependymomas, gross or near-total resection is typically followed by conformal focal radiotherapy (RT). Although prophylactic cranial irradiation was once standard, focal RT is now preferred, as most recurrences are local and PCI has not shown a survival benefit (5, 6, 79, 80). Broader radiation fields are reserved for patients with confirmed dissemination by imaging or CSF cytology. Limiting irradiated brain volume improves quality of life by reducing the risks of neurocognitive decline, stroke, and secondary malignancies.

Conformal RT targets the tumor bed with a margin while sparing healthy brain tissue. Standard doses range from 54 to 59.4 Gy; observational studies show no added benefit with higher doses after gross total resection (81), though escalation may be warranted for residual disease. Optimal margin size is under investigation, with trials such as ACNS0121 and ACNS0831 evaluating 1.0 cm and 0.5 cm clinical target volumes, respectively.

In the COG ACNS0121 phase II trial, 281 children with gross or near-total resection of grade 2 or 3 ependymomas (excluding completely resected supratentorial grade 2 tumors) received postoperative focal RT. At a median follow-up of 7.9 years, five-year event-free and overall survival rates were 69% and 86%, respectively. For patients with near-total or macroscopic resection (stratum 3), EFS and OS were 67% and 83%; for those with microscopic gross total resection (stratum 4), rates were 70% and 88% (11).

##### 7.1.1.2 Post-RT chemotherapy

Post-radiotherapy (RT) chemotherapy for ependymoma remains investigational pending mature data from the completed COG ACNS0831 trial. This phase III trial randomized children aged 1–21 years with newly diagnosed grade 2 or 3 ependymomas after gross-total or near-total resection to focal RT alone or RT with maintenance chemotherapy (vincristine, cisplatin, cyclophosphamide, and etoposide) (78). Preliminary results from 325 patients (median age, 4.9 years) at a median follow-up of 3.5 years demonstrated a trend towards improved event-free survival (EFS) with RT plus chemotherapy (78% vs. 72%; hazard ratio [HR], 0.73; 90% CI, 0.51–1.06). This benefit was statistically significant in a subgroup with complete or near-complete resections (81% vs. 71%, P = 0.03). Overall survival (OS) data remain unavailable.

Adherence to chemotherapy was suboptimal; 27% of the chemotherapy-assigned patients received RT alone, mostly due to refusal. An as-treated analysis, excluding patients who did not receive chemotherapy and some in the RT-alone arm who missed RT, showed improved EFS with chemotherapy (80% vs. 71%; HR, 0.58; 95% CI, 0.36–0.94), though this result is prone to bias. Final results of ACNS0831, including molecular subgroup outcomes, are pending. The ongoing SIOP-EP-II trial (NCT02265770), comparing adjuvant chemotherapy to observation post-resection and RT, will provide further clarity. Currently, adjuvant chemotherapy after RT is not recommended due to insufficient evidence of benefit.

#### 7.1.2 Subtotal resection, age 1 to 21 years

Incompletely resected grade 2 or 3 ependymomas are associated with inferior progression-free and overall survival compared to gross total resection. For these high-risk patients, current practice includes a short postoperative chemotherapy course, followed by second-look surgery when feasible, and then conformal radiotherapy (RT) (5, 12, 82). Active agents include cisplatin, carboplatin, cyclophosphamide, and etoposide, with greater efficacy seen in multidrug regimens (56, 77, 83–88). One to four cycles of chemotherapy are typically administered postoperatively, followed by MRI to reassess resectability. Patients with safely removable residual disease undergo second debulking prior to focal RT.

RT dosing mirrors that used in completely resected tumors, though higher doses may be appropriate for residual macroscopic disease. This multimodal strategy is supported by both single- and multicenter studies (5, 11, 77, 82, 89). The most robust prospective data come from stratum 2 of the COG ACNS0121 trial, which enrolled 64 patients with incomplete resection. All received chemotherapy, second-look surgery when feasible (achieved in 39%), and focal RT. At a median follow-up of 7.9 years, event-free and overall survival were 37% and 70%, respectively (11). Chemotherapy consisted of two cycles of carboplatin, cyclophosphamide, etoposide, and vincristine over seven weeks.

Post-RT chemotherapy appears to confer no additional benefit. In the ACNS0831 trial, patients who achieved complete response through induction chemotherapy or second-look surgery had similar outcomes whether treated with RT alone or RT plus maintenance chemotherapy (78). No benefit was observed in those with residual disease post-induction.

#### 7.1.3 Children <1 year of age

Although adjuvant radiotherapy (RT) is the standard of care for most ependymoma patients, its use in infants is limited due to concerns about developmental toxicity (42). For children under 1 year, adjuvant chemotherapy is recommended to delay RT. Those with residual tumor after chemotherapy may be considered for second-look surgery and should ideally be managed at tertiary care centers. In children over 1 year, the benefits of focal RT generally outweigh the risks. Chemotherapy in lieu of RT for patients aged 1 to 3 should be restricted to clinical protocols.

No randomized trials directly compare immediate postoperative RT with chemotherapy followed by deferred RT. However, several cooperative group studies have explored this approach in young children:

- In a cohort of 41 children under age 3 (one with disseminated disease), multiagent chemotherapy followed resection. Regimens included vincristine, methotrexate, and cyclophosphamide alternating with cisplatin and etoposide, or a shorter vincristine–etoposide–cyclophosphamide protocol (90). Twenty-nine experienced local progression (median, 9 months). Of 13 survivors, six avoided RT. Five-year progression-free survival (PFS), event-free survival (EFS), and overall survival (OS) were 27%, 26%, and 37%, respectively, with no significant cognitive differences between those who received or avoided RT.
- In a study of 73 children under 5 without dissemination, seven chemotherapy cycles followed maximal resection (84). At a median follow-up of 5 years, four-year OS was 59%, and five-year EFS was 22%. RT was required for recurrence in 49% at a median of 15 months.
- Among 89 children aged 3 years or younger treated with resection and chemotherapy, 80 had nondisseminated disease; 50 relapsed (91). At 6-year median follow-up, five-year EFS and OS were 42% and 63%, respectively, and RT was avoided in 42%.

High-dose chemotherapy with autologous stem cell rescue remains investigational. In one pilot study, five children under age 3 with anaplastic ependymoma (most with residual or disseminated disease) were treated with this approach. All reached age 3 without RT; only one experienced progression at a median follow-up of 45 months (92).

The long-term neurocognitive consequences of various treatment strategies in infants remain poorly characterized and represent a critical area for future research.

#### 7.1.4 Patients with disseminated disease

Leptomeningeal dissemination or spinal seeding from intracranial ependymoma portends a poor prognosis, though patients with ZFTA fusion–positive tumors may experience prolonged survival (53). Management should be individualized. Given the limited sensitivity of cerebrospinal fluid cytology in ependymoma, repeat sampling 10 to 14 days after surgery is recommended to confirm initial findings. Referral to a tertiary center is advisable when feasible.

Postoperative craniospinal irradiation (CSI) is generally indicated but carries greater developmental risk than focal radiotherapy. In children younger than three years, CSI is typically deferred in favor of chemotherapy, with or without focal radiation.

#### 7.1.5 Areas of uncertainty and ongoing trials

•Observation after gross total resection of supratentorial grade 2 tumors.

Retrospective data suggest that adjuvant radiotherapy (RT) may be omitted in selected patients with completely resected supratentorial grade 2 ependymomas, though this approach remains controversial (69, 93–95). As most recurrences are local, deferring RT aims to avoid early toxicities, with salvage surgery and RT reserved for recurrence. In the COG ACNS0121 phase II trial, 11 pediatric patients managed with observation alone had a five-year progression-free survival (PFS) of 61%, and all were alive at five years (11). However, the small sample size limited conclusions. While North American trials such as ACNS0831 have guided much of current practice, the ongoing European SIOP-Ependymoma II trial is expected to provide critical data on the role of post-radiotherapy chemotherapy and observation in stratified patient groups.

•Chemotherapy alone in children over 1 year.

The role of primary chemotherapy in children over 1 year remains undefined. As modern conformal radiation techniques reduce long-term toxicities, replacing RT with chemotherapy should be limited to clinical trials.

#### 7.1.6 Adults, age >21 years

Intracranial ependymoma is rare in adults, and no randomized trials guide management. Treatment relies on retrospective studies and extrapolation from pediatric protocols. As in children, the extent of initial resection is the strongest predictor of outcome, and maximal safe resection is recommended for all patients (95–98). Postoperative focal radiotherapy (RT) is indicated for localized grade 3 (anaplastic) tumors and for residual disease after resection of grade 2 tumors (74, 75, 99, 100). Craniospinal RT is reserved for disseminated disease confirmed by imaging or CSF cytology (99). Adjuvant chemotherapy offers no proven benefit in adults.

The benefit of adjuvant focal RT after gross total resection of grade 2 ependymomas remains uncertain. Current guidelines from the National Comprehensive Cancer Network (NCCN) and the European Association for Neuro-Oncology (EANO) recommend observation, supported by retrospective data showing no survival advantage with RT in this setting (75, 99, 101, 102). Nonetheless, some advocate postoperative RT even after complete resection—particularly when microscopic residual disease is suspected—citing its tolerability, the limited efficacy of salvage options, and the risks of recurrence, especially in the posterior fossa.

### 7.2 Other ependymal tumors

Subependymomas are often incidental findings in older adults and typically require no treatment unless symptomatic or enlarging. When resection is indicated, complete removal of large, symptomatic tumors is usually curative, underscoring their more indolent course relative to other ependymomas (103). Radiotherapy is generally reserved for unresectable or progressive lesions.

## 8 Follow-up and monitoring

Although most recurrences in pediatric ependymoma occur within five years of diagnosis, late progression can occur, supporting surveillance beyond this window (104).

### 8.1 Surveillance

Imaging practices vary, but a strategy consistent with the Children’s Oncology Group (COG) ACNS0831 trial is commonly adopted:

**Brain MRI**: Every 3–4 months for the first 3 years post-treatment, every 6 months from years 3 to 5, and annually for an additional 2–5 years thereafter in survivorship care.

**Spine MRI**: Performed annually with brain MRI, if symptoms suggest spinal involvement, or if brain imaging shows progression.

The RAPNO group recommends more frequent spine imaging (e.g., every other brain MRI), and combined brain/spine studies at each interval for patients with metastatic disease or 1q gain (64), though these guidelines remain unvalidated and primarily research-focused.

### 8.2 Survivorship

Long-term survivors of childhood CNS tumors are at risk for neurocognitive deficits, focal neurologic impairments, hearing loss, endocrine and growth disturbances, radiation necrosis, vasculopathy, and second malignancies (42, 105–112). Morbidity in adult survivors often includes fatigue, pain, numbness, and sleep disruption (113). The COG offers long-term follow-up guidelines for these patients (114).

## 9 Recurrent disease

The long-term prognosis for patients with recurrent ependymoma is poor, with most succumbing within years of relapse despite extended palliation. First recurrence is local in approximately two-thirds of cases, metastatic (within the CNS) in about 20%, or combined in 10–15% (115, 116). Although various treatment options exist, patients and caregivers should be informed of the dismal long-term outlook following recurrence or progression after surgery and radiotherapy (RT). Thoughtful treatment selection can optimize palliation and quality of life, though no single approach is standard or proven effective (117).

Outside clinical trials, management should be individualized, considering age, original and recurrent disease location, metastasis, prior therapy, and functional status. Beyond experimental treatments, several strategies have been employed:

### 9.1 Surgery

Aggressive resection may offer effective palliation in select cases. Chemotherapy, with or without RT, may reduce residual or recurrent tumor to facilitate reresection, aiming to delay progression and death (83, 86, 118).

### 9.2 Radiation

Reirradiation of recurrent ependymomas may improve outcomes, achieving salvage in select cases (117, 119–123). Options include stereotactic radiosurgery (SRS), focal fractionated reirradiation, or craniospinal irradiation (CSI), tailored to recurrence location, patient age, and extent (119, 124). Focal reirradiation carries a risk of disseminated metastases, with local recurrence eventually affecting most patients. In a large retrospective series of 101 patients undergoing aggressive repeat resection and reirradiation (median 27 months after initial RT), median progression-free survival (PFS) and overall survival (OS) from the start of second RT were 27 and 75 months, respectively (123). Tumor progression occurred in 57 patients (56%) after focal RT or CSI with boost, with local failure contributing in 35 (61%). Outcomes were best in patients with distant-only failure post-initial RT, no anaplasia at recurrence, and subsequent CSI with boost reirradiation. The completed phase II RERTEP trial (NCT02125786) investigated this approach, with results pending.

### 9.3 Chemotherapy

Conventional chemotherapy may provide symptomatic relief in recurrent ependymoma, though no standard regimen exists for children (99). Agents with the most activity—based on adjuvant or neoadjuvant data—include cisplatin, carboplatin, cyclophosphamide, and etoposide. In a series of 28 adults with recurrent or progressive intracranial disease, objective responses were observed in six patients, with better outcomes in those receiving platinum-based regimens (125). Modest activity has also been reported with oral etoposide, nitrosoureas, temozolomide, and fluorouracil (99, 126–131). However, responses are generally short-lived, with rapid disease progression following chemotherapy alone.

### 9.4 Molecular targets

Emerging molecular targets in ependymoma include EGFR (18), VEGF, and various epigenetic and metabolic regulators (132–136). In adults, temozolomide combined with lapatinib (a HER2/EGFR inhibitor) has shown activity in recurrent disease. A phase II trial involving 50 adults with recurrent intracranial or spinal ependymoma reported a median progression-free survival (PFS) of 7.8 months and overall survival (OS) of 2.25 years, with two complete and six partial responses; many experienced symptom stabilization or improvement (137). In a series of eight adults, six had partial responses to bevacizumab, with a median time to progression of six months (138), although other retrospective studies suggest limited efficacy (139).

In children, treatment remains investigational. A trial of bevacizumab and irinotecan in 13 children with recurrent or progressive ependymoma yielded no objective responses (median time to progression, 2.2 months) (140). Similarly, a phase II study of bevacizumab plus lapatinib in 24 children showed no benefit (141).

## 10 Prognosis

### 10.1 Children

Despite surgery and adjuvant therapy, pediatric intracranial ependymomas carry a poor long-term prognosis, with 10-year overall survival (OS) ranging from 50% to 75% (11, 77, 104, 142). Several factors influence disease-free survival following treatment (143):

#### 10.1.1 Extent of resection

Local recurrence accounts for approximately 80% of failures, underscoring the importance of maximal safe resection (66, 100, 144–146). Gross total resection is consistently associated with better outcomes, though benefit varies (5, 66, 100, 144). Still, up to 40% of children relapse or die within 10 years (104).

#### 10.1.2 Tumor location

Data on survival by location are mixed. Some studies report better outcomes for supratentorial tumors (5, 142, 147); others favor infratentorial lesions (100). Cervical spinal involvement is linked to poorer survival due to increased risk of distant relapse (148).

#### 10.1.3 Age

Older children fare better than infants, who often present with infratentorial PFA tumors and face delayed radiotherapy due to concerns over developmental toxicity (146).

#### 10.1.4 Histologic grade

The prognostic significance of histologic grade remains debated. Some studies associate grade 3 tumors with worse outcomes (11, 142, 149), but grading inconsistencies limit definitive conclusions (12).

#### 10.1.5 Molecular genetic groups

Molecular subgrouping may surpass histology in prognostic precision, with emerging classifications refining risk stratification.

#### 10.1.6 Additional molecular alterations

Chromosome 1q gain is associated with adverse outcomes in PFA tumors, confirmed by retrospective and prospective studies (15, 18, 24, 28, 44). Loss of 6q, especially when co-occurring with 1q gain, may define an ultra–high-risk subgroup (44, 46). In ZFTA fusion–positive tumors, *CDKN2A* deletion may also predict poor prognosis, though prospective validation is needed (45, 55, 56).

### 10.2 Adults

Intracranial ependymoma is uncommon in adults, and outcomes data remain limited. Two large European series (222 patients total) and one from a U.S. center (123 patients) reported 5-year survival rates of 67% to 85% and 10-year survival of 50% to 77% (96, 97, 150). Multivariate analyses associated poorer outcomes with high-grade histology, subtotal resection (151), tumor location (150, 152), and a Karnofsky Performance Status of 80 or lower (97). The detrimental effect of incomplete resection was corroborated in a population-based SEER study including both adults and children (68). A recent 2025 comprehensive review emphasizes the growing importance of molecularly guided management in adults, suggesting improved prognostic accuracy with molecular subgrouping, despite continued challenges due to disease heterogeneity and the rarity of cases in adults (153).

## 11 Summary of enhanced WHO classification of ependymomas

To consolidate the clinical, molecular, and prognostic features of ependymomas as classified under the enhanced WHO framework, **Table 1** provides a comprehensive summary of key subtypes across supratentorial and infratentorial regions, integrating data discussed throughout this review.

**Table 1:** Enhanced WHO classification of ependymomas: clinical and molecular features.

Location	Tumor classification	CNS WHO grade	Key molecular features	Common treatments	Prognosis	Epidemiology
Supra- tentorial	Supratentorial subependymoma	1	Typically no significant molecular abnormalities; occasional 1q gain or 6q loss (16, 24)	Maximal safe resection (GTR), observation; RT for symptomatic or residual tumors (74, 103)	Excellent, low recurrence rate, 10-year OS>90% (103)	Rare, predominantly in middle-aged/older males
	Supratentorial ependymoma, ZFTA fusion-positive	2, 3	ZFTA-RELA fusion (70-80%), CDKN2A biallelic loss indicates poor prognosis (45, 55)	Maximal safe resection (GTR/STR) + postoperative focal RT, chemotherapy for recurrence or residual disease (11, 74, 77)	Poor,high recurrence rate, 10-year OS 50-70%, worse with CDKN2A loss (55)	Common in children and adolescents, equal male:female ratio
	Supratentorial ependymoma, YAP1 fusion-positive	2, 3	YAP1fusion, limited molecular data available (12, 45)	Maximal safe resection (GTR/STR) + postoperative focal RT, chemotherapy for recurrence (11, 74, 77)	Potentially favorable, but requires further study; may be better in infants than ZFTA-fused tumors (12, 45)	Rare, predominantly in infants
	Supratentorial ependymoma, no ZFTA or YAP1 fusion	2, 3	Molecular heterogeneity, further research needed (57, 58)	Maximal safe resection (GTR/STR) + postoperative focal RT, chemotherapy for recurrence (11, 74, 77)	Uncertain prognosis, requires molecular subtyping studies (57, 58)	Rare,clinically and molecularly diverse
infra- tentorial	Posterior fossa subependymoma	1	Typically no significant molecular abnormalities; occasional 1q gain (16, 24)	Maximal safe resection (GTR), observation; RT for symptomatic or residual tumors (74, 103)	Excellent low recurrence rate, 10-year OS>90% 103]	Rare, predominantly in middle-aged/older males
	Posterior fossa ependymoma, group PFA*	2, 3	CpG island methylator phenotype, H3K27me3 loss, 1q gain/6q loss indicate poor prognosis (11, 24, 44)	Maximal safe resection GTR/STR)+postoperative focal RT, chemotherapy for infants or recurrence (42, 74, 77)	Poor, high-risk in children, 10-year OS 50-70% (11, 42), worse with 1q gain (24, 44)	Common in infants and young children, equal male:female ratio
	Posterior fossa ependymoma, group PFB*	2, 3	Stable molecular profile, no significant high-risk genetic markers (38, 41)	Maximal safe resection (GTR/STR)+postoperative focal RT, chemotherapy for recurrence (74, 77, 99)	Favorable, better prognosis in children and adults, 10-year OS 70-85% (41, 104)	Occurs in older children and adults, more common in older age groups

## 12 Conclusion

Intracranial ependymomas predominantly arise in early childhood (median age: 5 years), while spinal ependymomas are more frequently observed in adults. The spectrum includes subependymoma (WHO grade 1), myxopapillary ependymoma (WHO grade 2), and classic ependymoma localized to the supratentorial, posterior fossa, or spinal compartments. The traditional distinction between grade 2 and grade 3 tumors is increasingly being superseded by molecular stratification. Clinical symptoms vary by tumor location: posterior fossa lesions commonly present with headache, nausea, ataxia, and papilledema secondary to hydrocephalus.

Diagnosis requires histopathological confirmation and is guided by imaging features, tumor location, and patient age. Staging with spinal MRI and cerebrospinal fluid analysis is essential, as dissemination is present in approximately 10% of cases at diagnosis.

Maximal safe surgical resection remains the cornerstone of initial therapy, with gross total resection as the goal; however, proximity to eloquent structures such as the brainstem may preclude complete excision, necessitating referral to specialized pediatric neurosurgical centers. Adjuvant focal radiotherapy (RT) is the standard of care for children aged 1–21 years with WHO grade 2 or 3 ependymomas following gross or near-total resection, except in two key scenarios: children aged 1–3 years, where chemotherapy is administered within clinical protocols to defer RT, and patients with supratentorial grade 2 tumors, for whom observation may be appropriate. In cases of subtotal resection, management typically includes chemotherapy, second-look surgery (when feasible), and RT. Infants under 1 year are treated with chemotherapy to delay RT, while patients with disseminated disease require craniospinal irradiation or systemic chemotherapy. In adults, focal RT is commonly employed for grade 3 tumors or incompletely resected grade 2 lesions.

Surveillance should continue for 7–10 years post-treatment. Despite multimodal management, the 10-year overall survival rate for pediatric patients remains between 50% and 70%.
